# Selective Voltammetric Sensor for the Simultaneous Quantification of Tartrazine and Brilliant Blue FCF

**DOI:** 10.3390/s23031094

**Published:** 2023-01-17

**Authors:** Liliya Gimadutdinova, Guzel Ziyatdinova, Rustam Davletshin

**Affiliations:** 1 Analytical Chemistry Department, Kazan Federal University, Kremleyevskaya, 18, Kazan 420008, Russia; 2 Department of High Molecular and Organoelement Compounds, Kazan Federal University, Kremleyevskaya, 18, Kazan 420008, Russia

**Keywords:** voltammetric sensors, chemically modified electrodes, metal oxide nanomaterials, synthetic dyes, beverages, food analysis

## Abstract

Tartrazine and brilliant blue FCF are synthetic dyes used in the food, cosmetic and pharmaceutical industries. The individual and/or simultaneous control of their concentrations is required due to dose-dependent negative health effects. Therefore, the paper presents experimental results related to the development of a sensing platform for the electrochemical detection of tartrazine and brilliant blue FCF based on a glassy carbon electrode (GCE) modified with MnO_2_ nanorods, using anodic differential pulse voltammetry. Homogeneous and stable suspensions of MnO_2_ nanorods have been obtained involving cetylpyridinium bromide solution as a cationic surfactant. The MnO_2_ nanorods-modified electrode showed a 7.9-fold increase in the electroactive surface area and a 72-fold decrease in the electron transfer resistance. The developed sensor allowed the simultaneous quantification of dyes for two linear domains: in the ranges of 0.10–2.5 and 2.5–15 μM for tartrazine and 0.25–2.5 and 2.5–15 μM for brilliant blue FCF with detection limits of 43 and 41 nM, respectively. High selectivity of the sensor response in the presence of typical interference agents (inorganic ions, saccharides, ascorbic and sorbic acids), other food dyes (riboflavin, indigo carmine, and sunset yellow), and vanillin has been achieved. The sensor has been tested by analyzing soft and isotonic sports drinks and the determined concentrations were close to those obtained involving the chromatography technique.

## 1. Introduction

**Figure 1:**
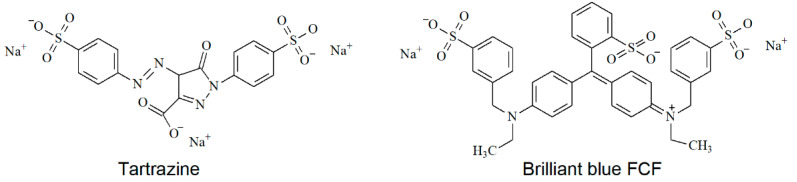
Structure of the synthetic dyes under consideration.

Tartrazine and brilliant blue FCF (Figure 1) are synthetic azo and triphenylmethane dyes, respectively, which are often used in the food, cosmetic, and pharmaceutical industries. Both dyes show dose-dependent negative health effects of various severity [1,2,3]. Tartrazine can stipulate carcinogenesis [2,4] as far as metabolized with the formation of the aromatic amines being well-known carcinogens. Therefore, the average daily intake of tartrazine and brilliant blue FCF is regulated at 7.5 mg/kg_bw_ [5] and 6 mg/kg_bw_ [6]. Therefore, strict control of their content in beverages, candies, jellies, ice cream, etc. [7] is required.

Various analytical techniques, such as chromatography [8,9,10,11,12], electrophoresis [13,14,15], or spectrophotometry [16,17,18] are usually applied for the determination of dye concentrations in food products. The possibility of simultaneous detection of several food dyes is the main advantage of chromatography and electrophoresis. Spectrophotometry is less selective and preliminary separation [16,17] or chemometric treatment [18] of the data is required to get reasonable results. Solid-phase extraction of dyes is often applied in chromatography [9,11], electrophoresis [14], and spectrophotometry [16,17]. An additional sample pretreatment step makes the procedure more tedious, increases the cost of the analysis, and can lead to a partial loss of analytes. Furthermore, organic solvents that are harmful to the environment are often used as eluents.

The direct determination of tartrazine and brilliant blue FCF is of practical interest. Electrochemical sensors are a good alternative to the methods mentioned above. Various chemically modified electrodes based on carbon nanomaterials, metal and metal oxide nanoparticles, polymeric coverages, and their combinations have been developed for the determination of tartrazine [19]. Brilliant blue FCF is a less-studied dye in electroanalytical chemistry. Two sensors based on multi-walled carbon nanotubes have been reported for the quantification of brilliant blue FCF in beverages [20,21].

Tartrazine and brilliant blue FCF are often used together for the production of green-colored foodstuffs and beverages. Therefore, voltammetric approaches for the simultaneous quantification of these dyes are also needed. Very few data have been presented in the literature related to the simultaneous electrochemical detection of tartrazine and brilliant blue FCF. Electrochemical sensors based on carbon black–polyethylene composite electrodes [22], ionic liquid-modified expanded graphite paste electrodes [23], and multi-walled carbon nanotube paste electrodes [24] have been reported so far. The corresponding list of merits is summarized in Table 1.

The application of metal oxide nanomaterials as electrode surface modifiers could be a prospective approach for the simultaneous quantification of tartrazine and brilliant blue. Nanostructures of TiO_2_, In_2_O_3_, CeO_2_, ZnO, Fe_3_O_4_, etc. are promising materials for bio- and electrochemical sensing due to the chemical and electrochemical inertness, large surface area, and biocompatibility [25,26]. In particular, voltammetric sensors based on metal oxide nanoparticles have been developed for the determination of antioxidants [27,28,29,30,31,32,33], pharmaceuticals [34] and drugs [35], and pollutants [36,37]. Few data are available for determining food dyes. Sensitive and selective sensors based on selenium dioxide nanoparticles have been developed for carminic acid [38] and indigo carmine [39] determination. Tartrazine quantification has been successfully achieved on the TiO_2_ [40,41] and CeO_2_ [42] nanoparticle-modified electrodes. Thus, the development of sensitive and selective voltammetric sensors based on metal oxide nanomaterials for the simultaneous quantification of tartrazine and brilliant blue FCF is of interest.

MnO_2_ nanomaterials (particles, wires, rods, needles, tubes, flowers, etc.) are widely studied and applied in chemistry and material science [43,44,45]. Their unique electrochemical properties, high charge transfer rate, large active surface area, non-toxic nature, and inexpensive cost are favorable for application in electrochemical sensors [46]. MnO_2_ nanomaterials show structural versatility, different oxidation states of Mn in these nanostructures, and the co-existence of two or three valence states in the same crystalline structure [45]. Furthermore, porous structure increases mass transport and provides a large surface area allowing improvement of target analyte response sensitivity. MnO_2_ nanorods-based voltammetric sensors for nitro-group containing organophosphates [47], nitroaromatic compounds [48], and sulfanilamide [49] have been recently developed. Sensing of rhodamine B [50] and quinoline yellow [51] has been achieved using composites of MnO_2_ nanorods with carbon nanomaterials. A dye’s analytical characteristics are acceptable for food analysis, although composite preparation is time-consuming. There are no electrodes modified with MnO_2_ nanorods for the simultaneous determination of food dyes to date. Furthermore, a simple, rapid, and reproducible procedure for electrode surface modification with MnO_2_ nanorods is required for the application of sensors in practice.

Among various dyes, the simultaneous determination of tartrazine and brilliant blue FCF has received limited attention to date although it is of high importance for food quality control. Therefore, the current work deals with the development of a voltammetric sensor for the simultaneous determination of tartrazine and brilliant blue FCF using a glassy carbon electrode (GCE) modified with MnO_2_ nanorods. Surfactants of various natures have been applied as dispersing agents to obtain a homogenous suspension of MnO_2_ nanorods. The stability of nanomaterial suspension in various surfactant media has been studied. Cationic cetylpyridinium bromide has been selected as a dispersive agent. Scanning electron microscopy (SEM) and electrochemical methods have been applied for the characterization of surface morphology and electrochemical properties of the sensor. The electrooxidation parameters of tartrazine and brilliant blue FCF have been found using cyclic voltammetry data. The applicability of the developed sensor in real samples analysis (soft and isotonic sports drinks) has been shown.

## 2. Materials and Methods

### 2.1. Reagents

Tartrazine (85% purity) was purchased from Sigma (St. Louis, MO, USA), and brilliant blue FCF (85% purity) from Sigma-Aldrich (Steinheim, Germany). Ascorbic acid (99% purity), sunset yellow (98% purity), vanillin (99% purity), sorbic acid (99% purity), riboflavin (98% purity), and indigo carmine (85% purity) were purchased from Aldrich, Steinheim, Germany and have been used in the interference study. 10 mM standard solutions for each compound were prepared in distilled water.

MnO_2_ nanorods (99%, diameter × *L* = 5–30 nm × 80–100 nm) from Sigma-Aldrich (Steinheim, Germany) were used as electrode surface modifiers. Sodium dodecyl sulfate of Ph. Eur. grade (Panreac, Barcelona, Spain), 98% cetylpyridinium bromide and Triton X-100 (Aldrich, Steinheim, Germany), and Brij^®^ 35 (Acros Organics, Geel, Belgium) were used as dispersing agent. Standard 1.0 mM surfactant solutions were prepared by dissolving the exact weight of the compound in distilled water. Thus, each dispersive agent was used separately.

1 mg mL^−1^ suspension of MnO_2_ nanorods was prepared in the corresponding dispersive agent by sonication for 40 min in an ultrasonic bath (WiseClean WUC-A03H (DAIHAN Scientific Co., Ltd., Wonju-si, Republic of Korea).

Other reagents were c.p. grade. The laboratory temperature was (25 ± 2 °C).

### 2.2. Equipment

Voltammetric and chronoamperometric measurements were performed using a potentiostat/galvanostat μAutolab Type III (Eco Chemie B.V., Utrecht, The Netherlands) and NOVA 1.7.8 software (Eco Chemie B.V., Utrecht, The Netherlands). Electrochemical impedance spectroscopy (EIS) experiments were performed involving potentiostat/galvanostat Autolab PGSTAT 302N with the FRA 32M module (Eco Chemie B.V., Utrecht, The Netherlands) and the NOVA 1.10.1.9 software (Eco Chemie B.V., Utrecht, The Netherlands).

A three-electrode glass cell of 10 mL was used for electrochemical measurements. A glassy carbon electrode (GCE) of 3 mm diameter (CH Instruments, Inc., Bee Cave, TX, USA), or a MnO_2_ nanorod-modified electrode was used as working electrode, and a platinum wire as an auxiliary electrode. Potentials were measured vs. an Ag/AgCl reference electrode.

The pH measurements were carried out using the “Expert-001” pH meter (Econix-Expert Ltd., Moscow, Russia) with a glassy electrode.

A MerlinTM high-resolution field emission scanning electron microscope (Carl Zeiss, Oberkochen, Germany) was applied for electrode surface morphology characterization and operated at 5 kV accelerating voltage and a 300 pA emission current.

### 2.3. Procedures

#### 2.3.1. Fabrication of the Modified Electrode

GCE surface was initially polished on 0.05 µm alumina slurry and rinsed with acetone and distilled water. To fabricate the modified electrode, 5 μL of MnO_2_ nanorod suspension was drop cast on the surface of the working electrode and air-dried for 10 min.

#### 2.3.2. Electrochemical Measurements

Voltammetric measurements were performed according to the following protocol. Supporting electrolyte (phosphate buffer (PB) of various pH) was added to the electrochemical cell and five scans were performed to achieve a stable voltammogram. Then, an aliquot of the tartrazine, brilliant blue FCF, or their mixture was added to the electrochemical cell and cyclic voltammograms were recorded from 0.6 to 1.2 V at a potential scan rate of 100 mV s^−1^. The total volume of the solution in the electrochemical cell consisting of supported electrolytes and analyte/analytes was equal to 4.0 mL.

Anodic differential pulse voltammetry was used for quantification purposes. Pulse parameters were optimized. The corresponding voltammograms were recorded in the potential range of 0.50–1.20 V with a scan rate of 20 mV s^−1^. Baseline correction in NOVA 1.7.8 software (Eco Chemie B.V., Utrecht, The Netherlands) was applied for the calculation of the oxidation currents.

Chronoamperometry was performed in the presence of 1.0 mM hexacyanoferrate(II) ions in 0.1 M KCl at 0.45 V for 75 s.

EIS investigations were carried out in the presence of a 1.0 mM mixture of hexacyanoferrate(II)/(III) ions in 0.1 M KCl in the frequency range of 10 kHz–0.04 Hz (12 points per order of magnitude) with an applied sine potential amplitude of 5 mV at a polarization potential of 0.23 V. The potential was calculated as a half-sum of the redox peaks of hexacyanoferrate(II)/(III) ions. Impedance spectra were presented as Nyquist plots. The fitting of the impedance data was performed using Randles’ equivalent circuit in NOVA 1.10.1.9 software.

#### 2.3.3. Beverages Analysis

Commercially available soft and isotonic sports drinks were used as real samples. Preliminary filtration was performed through a 0.45 µm pore size nylon membrane filter. Then, the electrochemical measurements were performed in phosphate buffer pH 7.0. 3.7 mL of phosphate buffer was placed in the electrochemical cell and five differential pulse voltammograms were recorded in the potential window of 0.50–1.20 V with a scan rate of 20 mV s^−1^ at the pulse amplitude of 75 mV and pulse time of 25 ms. Then, 300 μL of the sample was added and a differential pulse voltammogram was run under the same conditions.

Chromatographic measurements were performed according to the protocol detailed in [52].

#### 2.3.4. Statistical Treatment

Five replicates (three replicates for chromatography) were performed. A confidence level of 0.95 was used for the statistical treatment of the data obtained. All results were expressed as the average value ± coverage interval. Validation of the developed and independent methods was performed using *F*- and *t*-tests.

The detection limit was calculated as 3SD_a_/*b*, where SD_a_ was the standard deviation of the calibration graph intercept and *b*—the calibration graph slope.

Regression analysis was performed using the OriginPro 8.1 software (OriginLab, Northampton, MA, USA).

## 3. Results and Discussion

### 3.1. Voltammetric Behavior of Food Dyes on Bare GCE

**Figure 2:**
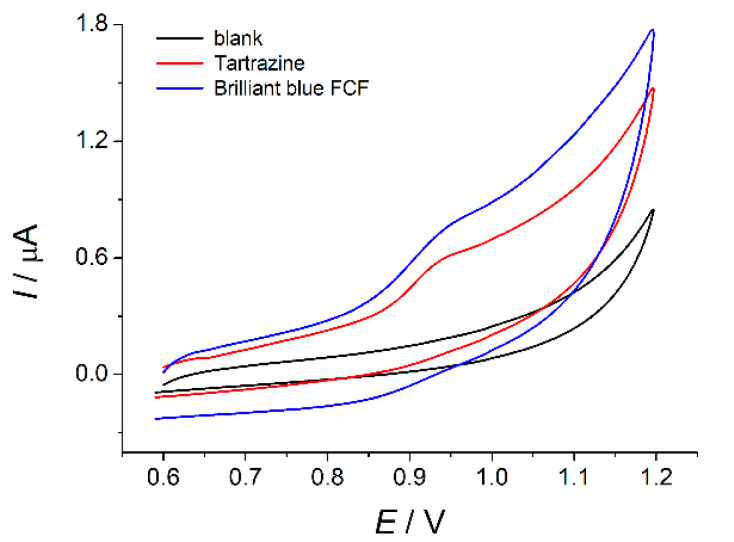
Cyclic voltammograms of 10 µM tartrazine and brilliant blue FCF on the bare GCE in phosphate buffer pH 7.0 (υ = 100 mV s^ −1^).

Tartrazine and brilliant blue FCF are oxidized at bare GCE in a neutral medium. There is one irreversible oxidation step at +0.94 V on the cyclic voltammograms of both dyes (Figure 2). Oxidation currents are also similar and equal to 70 ± 10 nA for a concentration of 10 µM. Such low oxidation currents are insufficient for successful dye quantification. Furthermore, dyes have the same oxidation potential on the bare GCE and their simultaneous detection and quantification are impossible. Electrode surface modification has to be used to solve these problems.

### 3.2. MnO_2_ Nanorods as Electrode Surface Modifier

#### 3.2.1. Selection of the Dispersing Agent for MnO_2_ Nanorods

MnO_2_ nanorods have been used as an electrode surface modifier. Surfactants have been shown as effective dispersing agents for the preparation of metal oxide nanoparticle dispersions [28,30,32,33,42]. Therefore, cationic, anionic, or non-ionic surfactant solutions have been studied as potential dispersing agents for MnO_2_ nanorods. Sonication for 40 min has been applied to achieve better dispersion of the nanomaterial. No stable suspensions of MnO_2_ nanorods (aggregation occurs after one-two hours) have been obtained using anionic sodium dodecyl sulfate and non-ionic Triton X-100 and Brij^®^ 35. On the contrary, cationic 1.0 mM cetylpyridinium bromide gives a homogeneous and stable suspension, which can be used for more than one month. This effect is caused by the electrostatic interaction of MnO_2_ nanorods bearing a partial negative charge in the neutral medium [53] with positively charged heads of cetylpyridinium bromide. The aggregates with positive zeta potential are formed. Similar behavior has been reported for MnO_2_ nanorods dispersed in polyethylenimine [54]. The electrostatic repulsion between these aggregates and cetylpyridinium bromide in solution provides stability of the suspension obtained.

#### 3.2.2. Characterization of MnO_2_ Nanorods-Based Electrode

**Figure 3:**
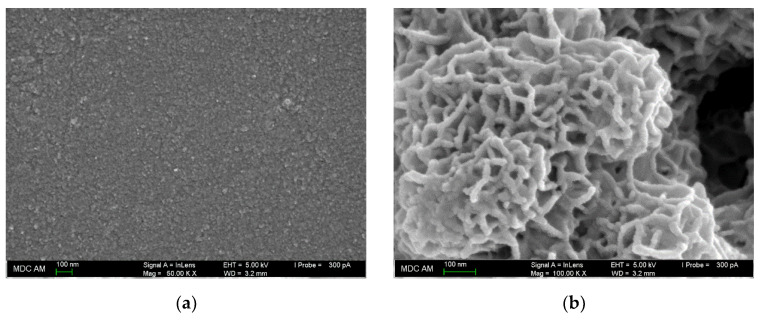
Surface morphology of the electrodes based on SEM: (**a**) bare GCE; (**b**) MnO_2_ nanorods-modified GCE.

Electrode surface morphology has been studied by SEM (Figure 3). The surface of the modified electrode is changed compared to bare GCE (Figure 3a). The MnO_2_ nanorods form a sponge-like structure consisting of intertwined nanorods with a width of 15–20 nm (Figure 3b). The roughness and area of the electrode surface are increased due to the porous structure with channels.

**Figure 4:**
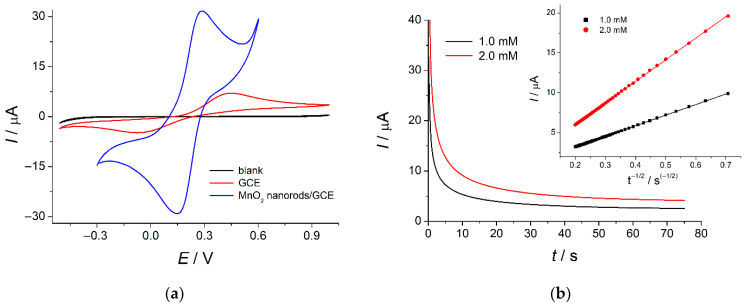
(**a**) Cyclic voltammograms of 1.0 mM hexacyanoferrate(II) ions in 0.1 M KCl on the bare GCE and MnO_2_ nanorods-modified GCE(υ = 100 mV s^−1^); (**b**) Chronoamperometric curves of hexacyanoferrate(II) ions in 0.1 M KCl on the bare GCE at 0.45 V. The inset is the plot of *I* vs. *t* ^−1/2^.

The electroactive surface area of the electrode has been calculated based on the oxidation of hexacyanoferrate(II) ions in 0.1 M KCl. Cathodic-to-anodic peak potential separation on the bare GCE indicates irreversible electrooxidation of the redox probe (Figure 4a). The electrochemical system reversibility is significantly improved on the modified electrode (Figure 4a). The increase in the electron transfer rate is caused by the electrostatic interaction of negatively charged hexacyanoferrate(II) ions with positively charged cetylpyridinium cations on the electrode surface. Similar results have been reported for metal oxide nanoparticle-modified electrodes [28,30,33].

The electroactive surface area of the bare GCE has been calculated using slopes of the *I* vs. *t*^−1/2^ plots (Figure 4b insert) obtained by chronoamperometry according to the Cottrell equation (Equation (1)) [55]. This approach is useful in the case of irreversible electrochemical systems.

(1) I=nFAcD12π−12t−12

where *I* is the oxidation current (A), the *n*—number of electrons participating in the oxidation of hexacyanoferrate(II) ions, *F*—the Faraday constant (96485 C mol^−1^), *A*—the electrode surface area (cm^2^), *c*—concentration of hexacyanoferrate(II) ions (mol cm^–3^), and *D*—diffusion coefficient of hexacyanoferrate(II) ions (cm^2^ s^–1^), *t*—time (s). For hexacyanoferrate(II) ions in 0.1 M KCl, *T* = 298 K, *n* = 1, *D* = 7.6 × 10^–6^ cm^2^ s^–1^ [55].

The Randles–Ševčík equation (Equation (2)) and the cyclic voltammetry data have been used for the modified electrode as far as the electrooxidation of hexacyanoferrate(II) ions proceeds reversibly.

(2) Ip=π12χpn32F32ACD12(RT)−12υ12

where *I_p_* is a peak current (A), *χ_p_*—normalized current for sweep experiments with a reversible system, *n*—the number of electrons, *F*—the Faraday constant (C mol^−1^), *A*—the electrode surface area (cm^2^), *C*—concentration (mol cm^–3^), *D*—diffusion coefficient (cm^2^ s^–1^), *R*—the gas constant (J mol^–1^ K^–1^), *T*—temperature (K), and υ—potential scan rate (V s^–1^) [55]. The electroactive surface area has been calculated from the slope of the plot *I*_ox_ vs. υ^½^ (Figure S1).

Thus, the MnO_2_ nanorods-modified electrode shows a 7.9-fold increase in the electroactive surface area compared to bare GCE (70 ± 2 mm^2^ vs. 8.9 ± 0.3 mm^2^, respectively).

**Figure 5:**
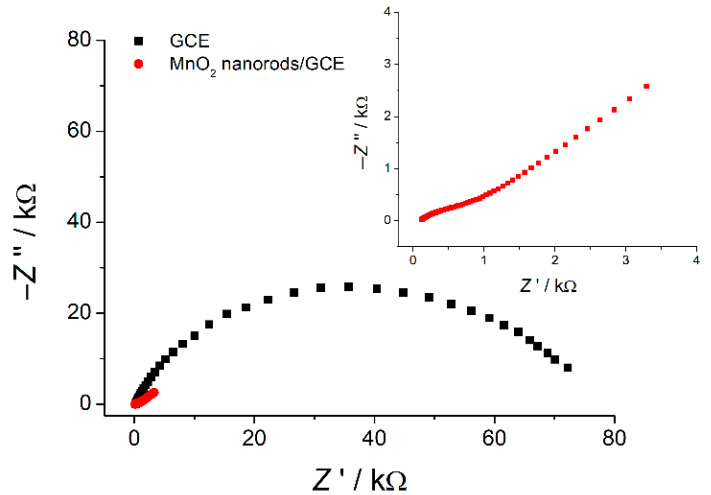
Nyquist plot for bare GCE and MnO_2_ nanorods/GCE in the presence of 1.0 mM hexacyanoferrate(II)/(III) ions in 0.1 M KCl. *E* = 0.23 V; frequency range = 10 kHz–0.04 Hz; amplitude = 5 mV.

Electron transfer properties of the electrodes have been studied by EIS. The corresponding Nyquist plots are presented in Figure 5.

Impedance spectra fitting has been performed using the Randles’ circuit (*R*_s_(*R*_et_*Q*) for bare GCE and *R*_s_(*Q*[*R*_et_*W*]) for modified electrode, where *R*_s_ is the electrolyte resistance, *R*_et_—the electron transfer resistance, *Q*—the constant phase element, and *W*—the Warburg impedance [56]). The results obtained are shown in Table 2. The modified electrode shows 72-fold lower electron transfer resistance that confirms the effectivity of the MnO_2_ nanorods in electron transfer reactions and, consequently, as an electrode surface modifier. The constant phase element is increased 29-fold compared to bare GCE which is caused by the following reasons:

- The porous structure of the modified electrode surface is indirectly confirmed by the heterogeneity factor *n* value [56];
- The increase in the total surface charge is due to the presence of cationic surfactant at the electrode surface.

The electrochemical data clearly indicate prospects of MnO_2_ nanorods as electrode surface modifiers providing large surface area and high electron transfer rate.

### 3.3. Cyclic Voltammetry of Dyes on the MnO_2_ Nanorods-Based Electrode

#### 3.3.1. Voltammetric Characteristics of Dyes

**Figure 6:**
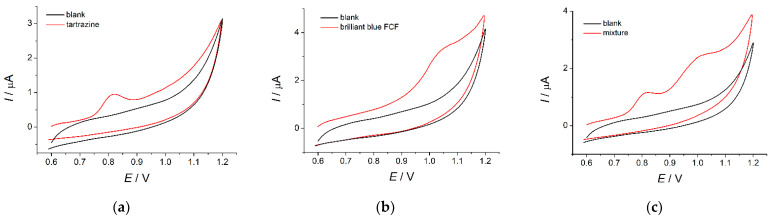
Cyclic voltammograms of 10 µM dyes on the MnO_2_ nanorods-modified electrode in phosphate buffer pH 7.0: (**a**) tartrazine; (**b**) brilliant blue FCF; (**c**) equimolar mixture of tartrazine and brilliant blue FCF. υ = 100 mV s ^−1^.

The voltammetric behavior of tartrazine and brilliant blue FCF on the MnO_2_ nanorods-modified electrode is significantly different compared to bare GCE (Figure 6a,b). Tartrazine oxidation potential is shifted to less positive values on 130 mV vs. bare GCE due to the electrocatalytic properties of MnO_2_ nanorods. An anodic shift of 80 mV in the oxidation potential of brilliant blue FCF is observed, which is probably caused by the structural properties of the dye. Oxidation currents of both dyes on the modified electrode are enhanced (six-fold) compared with those on the bare GCE due to an increase in the electroactive surface area of the modified electrode. Furthermore, preconcentration of dyes at the electrode surface can occur. The improvement in the shape of dyes’ voltammograms by using a modified electrode is also achieved.

Tartrazine and brilliant blue FCF peak potential separation on the MnO_2_ nanorods-modified electrode allows simultaneous detection of dyes as shown on the equimolar mixture. Two well-resolved oxidation peaks at 0.81 and 0.99 V have been observed on the cyclic voltammograms (Figure 6c). A peak potential separation of 180 mV in cyclic voltammetry is enough for the simultaneous determination of dyes.

#### 3.3.2. Effect of pH on the Tartrazine and Brilliant Blue FCF Response

The effect of phosphate buffer pH in the range of 5.0–8.0 on the voltammetric characteristics of dyes has been studied using cyclic voltammetry. Both dyes are irreversibly oxidized in the whole pH range which agrees well with research reports for other modified electrodes [20,21,42,57,58].

**Figure 7:**
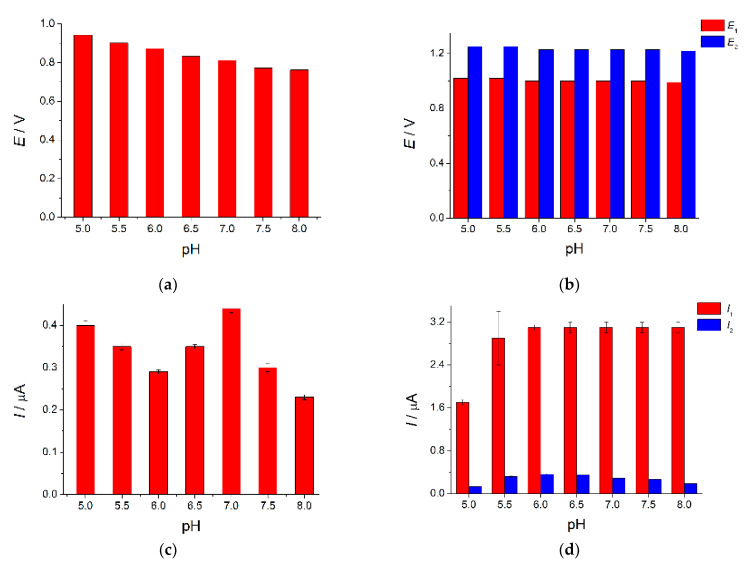
Effect of phosphate buffer pH on the voltammetric characteristics of dyes on the MnO_2_ nanorods-modified electrode: (**a**) oxidation potential of 10 µM tartrazine; (**b**) oxidation potentials of 100 µM brilliant blue FCF; (**c**) oxidation currents of 10 µM tartrazine; (**d**) oxidation currents of 100 µM brilliant blue FCF.

The voltammetric characteristics of the dyes are changed as the pH increases (Figure 7). The shift of tartrazine oxidation potential to less positive values with increasing pH (Figure 7a) confirms the participation of protons in the electrode reaction. Brilliant blue FCF shows two oxidation steps on the voltammograms. The second step is weakly pronounced and appears at high concentrations of dye. Similar behavior has been previously reported in [20]. Both oxidation potentials of brilliant blue FCF are shifted on the 20 mV to less values when pH is changed from 5.5 to 6.0 and then become pH-independent (Figure 7b). This behavior is explained by dye ionization (p*K*a equal to 5.63 and 6.58 [59]). Brilliant blue FCF exists in the anionic form in a neutral medium. Thus, its electrooxidation at pH 6.0–8.0 proceeds without proton transfer which is in line with data obtained on multi-walled carbon nanotubes-modified electrodes [24]. Tartrazine and brilliant blue FCF peak potential separation is significantly decreased in acidic media making their simultaneous determination impossible.

Tartrazine oxidation currents are decreased in the pH range of 5.0–6.0 and then start to grow to achieve a maximum at pH 7.0 (Figure 7c) which is probably caused by the ionization of tartrazine. The trianionic form of tartrazine exists at pH 5.0 insofar as the p*K*a of the sulfonic group is 2.0 and the p*K*a of the carboxylate group is 5.0 [60]. The first step oxidation currents of the brilliant blue FCF are increased with the pH growth achieving a maximum at pH 6.0 (Figure 7d). No changes in the oxidation currents have been observed in neutral and basic media. Oxidation currents in the second step are grown up to pH 6.5 and then gradually decreased. The first oxidation step has been used for characterization and analytical purposes.

Phosphate buffer pH 7.0 has been used in further investigations since both dyes can be determined simultaneously with sufficient sensitivity.

#### 3.3.3. Electrooxidation Parameters of Tartrazine and Brilliant Blue FCF

**Figure 8:**
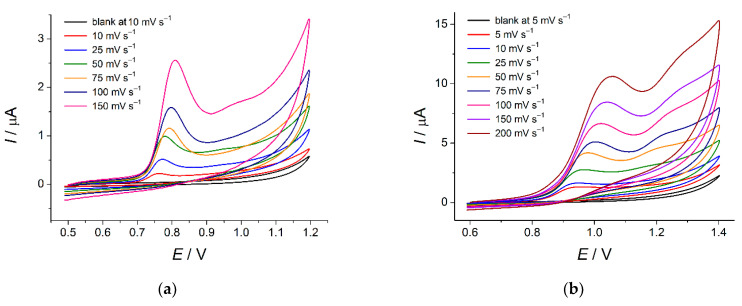
Cyclic voltammograms of dyes on the MnO_2_ nanorods-modified electrode in phosphate buffer pH 7.0 at various potential scan rates: (**a**) 30 µM of tartrazine; (**b**) 100 µM of brilliant blue FCF.

The potential scan rate effect on the voltammetric response of tartrazine and brilliant blue FCF has been evaluated (Figure 8).

Oxidation of tartrazine and brilliant blue FCF proceeds irreversibly as confirmed by the absence of cathodic steps and the anodic shift of oxidation potentials with the potential scan rate increase (Equations (3) and (4) for tartrazine and brilliant blue FCF, respectively).

*E* [V] = (0.857 ± 0.005) + (0.029 ± 0.003)lnυ [V s^−1^] *R*^2^ = 0.9809, (3)

*E* [V] = (1.090 ± 0.007) + (0.041 ± 0.003)lnυ [V s^−1^] *R*^2^ = 0.9840. (4)

The electrooxidation of tartrazine is a surface-controlled process, as confirmed by the linear plot of *I* vs. ν (Figure S2a) and a slope of 0.96 for the plot ln*I* vs. lnυ (Equations (5) and (6), respectively).

*I* [µA] = (0.03 ± 0.03) + (0.0119 ± 0.0003)υ [mV s^−1^] *R*^2^ = 0.9964, (5)

ln*I*[µA] = (2.41 ± 0.08) + (0.96 ± 0.02)lnυ [V s^−1^] *R*^2^ = 0.9968 (6)

Thus, the adsorption of tartrazine on the electrode surface occurred due to the electrostatic interaction between the positively charged surfactant and negatively charged at pH 7.0 tartrazine.

The diffusion-driven process for brilliant blue FCF electrooxidation has been confirmed by linear plot *I* vs. ν^½^ (Figure S2b) and the slope of 0.6 for the plot ln*I* vs. lnυ (Equations (7) and (8), respectively).

*I* [µA] = (−0.29 ± 0.09) + (0.34 ± 0.01)υ½ [mV s^−1^] *R*^2^ = 0.9943, (7)

ln*I* [µA] = (2.49 ± 0.04) + (0.60 ± 0.01)lnυ [V s^−1^] *R*^2^ = 0.9977. (8)

The electrooxidation parameters of tartrazine and brilliant blue FCF have been calculated using standard approaches for the surface- [61] and diffusion-driven processes [55,62] (Equations (S1)–(S6)). The corresponding data are summarized in Table 3.

**Scheme 1:**
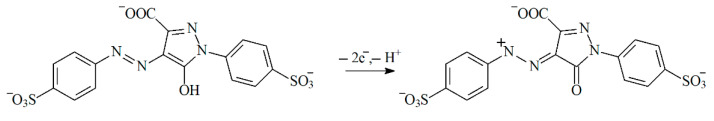
Electrooxidation of tartrazine.

Thus, tartrazine undergoes two-electron oxidation with protons participation (Scheme 1) that agrees well with data for carbon paste electrodes modified by silica impregnated with cetylpyridinium chloride [63] and by nanogold [64].

**Scheme 2:**
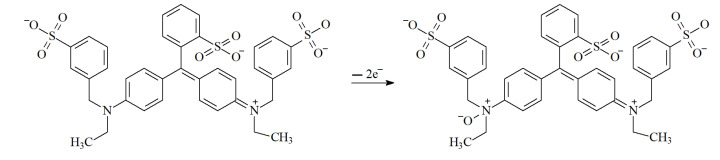
Electrooxidation of brilliant blue FCF.

The diffusion coefficient of brilliant blue FCF is almost twice higher than for the multi-walled carbon nanotube-modified carbon paste electrode [24]. These data confirm once more the effectivity of the electrode developed for brilliant blue FCF detection. The electrooxidation of brilliant blue FCF probably proceeds via the oxidation of the tertiary amine fragment with the formation of amine oxide that is quite stable in aqueous media (Scheme 2).

### 3.4. Voltammetric Sensor for the Simultaneous Quantification of Dyes

#### 3.4.1. Analytical Performance of the Sensor

The MnO_2_ nanorods-modified electrode has been used as a sensor for tartrazine and brilliant blue FCF using differential pulse voltammetry in phosphate buffer pH 7.0.

The effect of pulse parameters on the response of the dyes’ mixture has been evaluated. Pulse amplitude in the range of 25–100 mV and pulse time of 25–100 ms have been applied. Peak potential separation and oxidation currents of tartrazine and brilliant blue FCF have been considered (Figure S3). As can be seen from Figure S3a, the peak potential separation of the dyes is changed statistically insignificantly. The maximum of 200 mV has been achieved at a pulse amplitude of 75 mV and a pulse time of 25 ms. The oxidation currents of tartrazine and brilliant blue FCF are increased with the growth of pulse amplitude up to 75 mV (Figure S3b,c). Further increase of pulse amplitude to 100 mV leads to a decrease of the dyes’ oxidation currents. A similar trend is obtained with the increase in pulse time (Figure S3b,c). The best response of the tartrazine and brilliant blue FCF mixture has been observed at a pulse amplitude of 75 mV and a pulse time of 25 ms.

**Figure 9:**
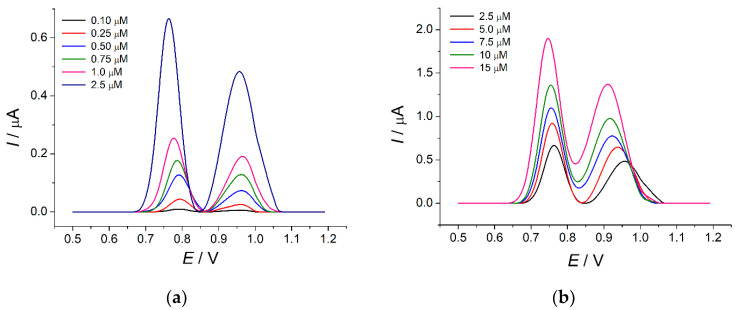
Baseline-corrected differential pulse voltammograms of equimolar mixtures of tartrazine and brilliant blue FCF on the MnO_2_ nanorods-based sensor in phosphate buffer pH 7.0: (**a**) concentration range is 0.10–2.5 µM; (**b**) concentration range is 2.5–15 µM. Pulse amplitude = 75 mV, pulse time = 25 ms, υ = 20 mV s^−1^.

Two well-resolved oxidation peaks at 0.77 and 0.97 V for tartrazine and brilliant blue FCF, respectively, have been observed on the differential pulse voltammograms (Figure 9). The oxidation currents increase linearly with the growth of the dye concentration in the ranges of 0.10–2.5 and 2.5–15 µM for tartrazine and 0.25–2.5 and 2.5–15 µM for brilliant blue FCF (Figure S4). The detection limits of 43 and 41 nM for tartrazine and brilliant blue FCF, respectively, have been achieved. The calibration graph parameters are presented in Table 4. The corresponding slopes indicate the high sensitivity of the developed sensor.

The analytical characteristics obtained are comparable to or worse than those reported for other modified electrodes (Table 1). Nevertheless, the sensor developed is easier to fabricate. The use of the anodic electrochemical window excludes the application of the inert atmosphere in the electrochemical cell which simplifies the analytical procedure. Furthermore, both dyes can be quantified in one voltammetric scan at the same pH. These are important advantages over the method [22] where, in fact, each dye is determined separately (reduction of tartrazine at pH 2.0 and brilliant blue FCF at pH 10.0). The most impressive detection limits are achieved using stripping mode [23]. However, it is based on the adsorptive preconcentration of the dyes for 500 s that increases the measurement time and can lead to the co-preconcentration of other components of the sample.

Independent oxidation of tartrazine and brilliant blue FCF in the first linear range has been confirmed by investigation of their non-equimolar mixtures (Figure S5). Moreover, the oxidation currents coincide with those for the equimolar mixtures. Therefore, calibration graphs for the first concentration range for equimolar mixtures are universal and can be used for individual and simultaneous quantification of tartrazine and brilliant blue FCF. A simple dilution can be applied in the case of the high content of dyes in real samples.

The precision of the developed method has been shown on the model mixtures of colorants at five concentration levels (Table 5).

The relative standard deviation values do not exceed 3.3%, confirming the absence of random errors in the determination and the high reproducibility of the electrode response (the electrode was renewed before each measurement). The recovery value is in the range of 99–100%.

#### 3.4.2. Selectivity Study

**Figure 10:**
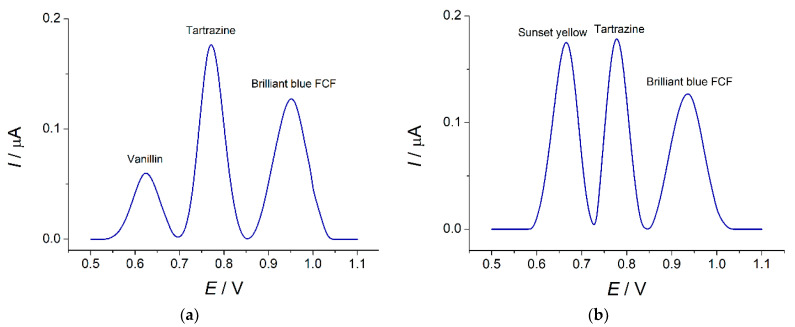

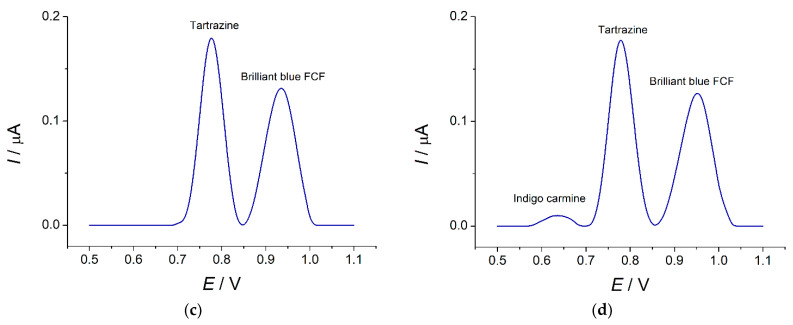
Baseline-corrected differential pulse voltammograms of ternary mixtures on the MnO_2_ nanorods-based sensor in phosphate buffer pH 7.0: (**a**) 0.75 µM vanillin, tartrazine, and brilliant blue FCF; (**b**) 0.75 µM sunset yellow, tartrazine, and brilliant blue FCF; (**c**) 37.5 µM riboflavin, 0.75 µM tartrazine and brilliant blue FCF; (**d**) 7.5 µM indigo carmine, 0.75 µM tartrazine and brilliant blue FCF. Pulse amplitude = 75 mV, pulse time = 25 ms, υ = 20 mV s^−1^.

Foodstuffs are characterized by a multi-component composition that can affect the response of dyes. The sensor selectivity has been tested on a 0.75 µM mixture of tartrazine and brilliant blue FCF. Inorganic ions (K^+^, Mg^2+^, Ca^2+^, NO_3_^−^, Cl^−^, and SO_4_^2−^) and saccharides (glucose, rhamnose, and sucrose) are electrochemically inactive in the anodic potential range considered and do not affect the sensor response to target dyes at 1000- and 100-fold excess, respectively. Other organic compounds used as preservatives, aromatic additives, and dyes in the food industry have been studied. Ascorbic acid is oxidized at 0.29 V, but it does not affect tartrazine and the brilliant blue FCF response up to a 10-fold excess. Sorbic acid is electrochemically silent on the voltammograms and does not show an interference effect. Vanillin shows a well-defined oxidation peak at 0.62 V, which does not overlap with the oxidation peaks of tartrazine and brilliant blue FCF up to an equimolar ratio (Figure 10a).

Other food dyes (sunset yellow, indigo carmine, and riboflavin) are also electrochemically active at the MnO_2_ nanorods-modified electrode. Sunset yellow is oxidized at 0.67 V. The variation of the sunset yellow concentration in ternary mixtures with tartrazine and brilliant blue FCF has shown the absence of an interference effect up to equimolar concentrations (Figure 10b). The partial overlap of the oxidation peaks and current redistribution take place at a higher concentration of sunset yellow.

A weakly pronounced oxidation step at 0.66 V has been observed on the voltammograms of riboflavin. Its addition to the mixture of tartrazine and brilliant blue FCF leads to an insignificant increase in tartrazine oxidation currents. Nevertheless, riboflavin at concentrations up to 37.5 µM does not give an oxidation step on the voltammograms of the ternary mixture (Figure 10c). Therefore, a 50-fold excess of riboflavin does not interfere with the response of tartrazine and brilliant blue FCF.

Indigo carmine is oxidized at 0.35 and 0.64 V. The weak oxidation step at 0.64 V has been observed in the electrochemical window of 0.5–1.2 V that is used for the quantification of tartrazine and brilliant blue FCF. The oxidation currents are negligible low (10 ± 2 nA for 7.5 µM concentration). Three well-resolved oxidation peaks are obtained for the ternary mixture confirming the selectivity of tartrazine and brilliant blue FCF detection in the presence of 10-fold excess of indigo carmine (Figure 10d).

Thus, the high selectivity of the sensor developed towards tartrazine and brilliant blue FCF is an important advantage over other electrodes [22,23,24].

#### 3.4.3. Repeatability, Reproducibility and Robustness of the Sensor

Significantly lower oxidation currents of tartrazine and brilliant blue FCF have been observed on the second scan caused by the adsorption of oxidation products at the MnO_2_ nanorods-modified electrode. Therefore, the sensor surface has been mechanically renewed after each measurement (see Section 2.3.1). Thus, a relative standard deviation of 0.71–3.3% for the twenty-five electrodes at the five concentration levels indicates perfect reproducibility of the sensor response. 

The robustness of the developed sensor has been evaluated. The change of the electrodes to other ones of the same type, orthophosphoric and sodium hydroxide used for the phosphate buffer preparation provides the relative standard deviation of the tartrazine and brilliant blue FCF determination of 1.0–3.5%. Thus, the robustness of the sensor developed is sufficient.

#### 3.4.4. Real Samples Analysis

The practical applicability of the sensor developed has been tested on soft and isotonic sports drinks. One of them (sample 1) contains only brilliant blue FCF. Other samples (No 2–4) contain both dyes. Nevertheless, brilliant blue FCF content (0.25–1.66 mg L^−1^ according to chromatography data) is too low to be detected by voltammetry taking into account the dilution of the sample. Typical voltammograms for the drinks are shown in Figure S6. There are well-defined oxidation peaks at 0.76 and 0.97 V corresponding to the dyes under consideration, which is confirmed by the standard addition method (Figure S6a for brilliant blue FCF and Figure S6b for tartrazine). Recovery values of 99–100% (Table S1) confirm the absence of matrix effects and the applicability of the sensor in real samples analysis.

The quantification of dyes in soft and isotonic sports drinks is shown in Table 6. Voltammetric data have been compared with chromatographic determination [52]. Results of the one-sample *t*-test confirm the absence of systematic errors in tartrazine and brilliant blue FCF determination using the developed sensor. *F*-test indicates similar precision of voltammetry and chromatography.

## 4. Conclusions

A MnO_2_ nanorods-based voltammetric sensor has been developed for the first time for the determination of food colorants. Simultaneous determination of tartrazine and brilliant blue FCF has been achieved using a created sensor. Cationic surfactant cetylpyridinium bromide has been used as a dispersing agent providing a stable suspension of MnO_2_ nanorods. Well-resolved oxidation peaks of tartrazine and brilliant blue FCF (Δ*E* = 180 mV in cyclic voltammetry) have been obtained on MnO_2_ nanorods-modified electrodes instead of fully overlapped signals at the bare GCE. The oxidation currents of both dyes are increased six-fold due to the porous structure of the electrode surface with a large electroactive area. Electrochemical data confirm a significant increase in the electron transfer rate for the MnO_2_ nanorods-based electrode vs. bare GCE. Therefore, the developed sensor is a promising tool in electroanalysis.

The MnO_2_ nanorods-based sensor shows improved analytical and operational characteristics in the direct simultaneous determination of tartrazine and brilliant blue FCF. The method developed excludes the preconcentration step, making determination less tedious. Another important advantage of the developed sensor is the high selectivity of the response to target colorants in the presence of a wide range of interferences including inorganic ions, saccharides, organic acids, aromatic additives, and other colorants. Furthermore, the sensor is easy to fabricate and is robust. The versatility of the developed sensor has been confirmed by real samples analysis. The results obtained significantly expand the analytical capabilities of electrochemical sensors in food safety and quality control.

## Figures and Tables

**Table 1:** List of merits of electrochemical sensors for the simultaneous determination of tartrazine and brilliant blue FCF.

Sensor	Method	Dye	Redox Process	Detection Limit (µM)	Linear Dynamic Range (µM)	Ref.
Carbon ink film-modified carbon black-polyethylene composite electrode	First-order derivative LSV ^1^	Tartrazine	Reduction	0.019	0.037–1.38	[22]
		Brilliant blue FCF		0.011	0.025–2.52	
1-Butyl-3-methylimidazolium hexafluorophosphate-modified expanded graphite paste electrode	Square-wave stripping voltammetry	Tartrazine	Oxidation	0.0033	0.010–1.0	[23]
		Brilliant blue FCF		0.002	0.0050–0.10; 0.10–4.0	
Multi-walled carbon nanotubes-carbon paste electrode	Differential pulse voltammetry	Tartrazine	Oxidation	–	0.1–30	[24]
		Brilliant blue FCF		–	0.1–24	

^1^ Linear sweep voltammetry.

**Table 2:** Fitted parameters of the electrochemical impedance (*n* = 5; *p* = 0.95).

Electrode	*R*_s_ (Ω)	*R*_et_ (kΩ)	*Q* (µΩ^−1^)	*n*	*W* (µΩ^−1^)	χ^2^
GCE	245 ± 5	72 ± 3	3.7 ± 0.2	0.789	–	0.028
MnO_2_ nanorods/GCE	98 ± 3	1.0 ± 0.2	108 ± 6	0.507	440 ± 10	0.031

**Table 3:** Electrooxidation parameters of colorants on the MnO_2_ nanorods-modified electrode.

Dye	Electrode Reaction Nature	α_a_^1^	*n* ^2^	*D* (cm^2^ s^−1^) ^3^	*Г* (pmol cm^−2^) ^4^	*k*_s_ (s^−1^) ^5^	*k*^0^ (cm s^−1^) ^6^
Tartrazine	Surface control	0.50	1.8		152 ± 4	864	
Brilliant blue FCF	Diffusion	0.42	2	(5.1 ± 0.8) × 10^−5^			(3.7 ± 0.4) × 10^−3^

^1^ Anodic transfer coefficient. ^2^ Number of electrons transferred. ^3^ Diffusion coefficient. ^4^ Surface coverage. ^5^ Heterogenous electron transfer rate constant. ^6^ Standard heterogeneous electron transfer rate constant.

**Table 4:** Calibration graphs parameters for tartrazine and brilliant blue FCF.

Dye	Linear Dynamic Range (µM)	*I* = *a* + *bc*		
		*a* ± SD (µA)	(*b* ± SD) × 10^−4^ (µA M^−1^)	*R* ^2^
Tartrazine	0.10–2.5	−0.024 ± 0.004	28.0 ± 0.3	0.9994
	2.5–15	0.49 ± 0.02	7.8 ± 0.2	0.9965
Brilliant blue FCF	0.25–2.5	−0.033 ± 0.003	22.1 ± 0.2	0.9996
	2.5–15	0.420 ± 0.005	4.02 ± 0.05	0.9993

**Table 5:** Simultaneous quantification of tartrazine and brilliant blue FCF in model mixtures using MnO_2_ nanorods-based sensor in phosphate buffer pH 7.0 (*n* = 5; *p* = 0.95).

Added (µM)	Tartrazine			Brilliant Blue FCF		
	Found (µM)	RSD (%)	*R* (%)	Found (µM)	RSD (%)	*R* (%)
0.25	0.25 ± 0.01	3.3	100 ± 3	0.25 ± 0.02	2.9	100 ± 3
0.75	0.75 ± 0.03	1.4	100 ± 4	0.74 ± 0.02	1.0	99 ± 3
2.5	2.51 ± 0.09	1.4	100 ± 4	2.5 ± 0.1	1.8	100 ± 4
7.5	7.5 ± 0.1	1.0	100 ± 1	7.53 ± 0.08	0.71	100 ± 1
15	15.0 ± 0.5	1.9	100 ± 3	14.9 ± 0.5	1.3	99 ± 3

**Table 6:** Quantification of tartrazine and brilliant blue FCF in soft and isotonic sports drinks (*p* = 0.95).

Dye	Sample	Dye Contents (mg L^−1^)				*t*-Test ^1^	*F*-test ^2^
		Voltammetry	RSD (%)	Chromatography	RSD (%)		
Tartrazine	1	4.6 ± 0.1	2.1	4.7 ± 0.2	1.6	2.31	1.67
Brilliant blue FCF	2	12.7 ± 0.3	2.0	13 ± 1	3.0	2.29	2.39
	3	5.5 ± 0.1	1.6	5.5 ± 0.2	1.6	0.152	1.01
	4	3.84 ± 0.09	1.8	3.88 ± 0.05	0.52	0.949	12.0

^1^*t*_crit_ = 2.45 at α = 0.05 and *f* = 6. ^2^ *F*_crit_ = 19.25 at α = 0.05 and *f*_1_ = 4, *f*_2_ = 2.

## Data Availability

The data presented in this study are available in the electronic Supplementary Materials.

## Supplementary Materials

The following are available online at https://www.mdpi.com/article/10.3390/s23031094/s1, Figure S1: Plot *I*_ox_ vs. υ^½^ for the electrooxidation of hexacyanoferrate(II) ions on the MnO_2_ nanorods-modified GCE in 0.1 M KCl, Figure S2: Effect of potential scan rate on the oxidation currents of dyes on the MnO_2_ nanorods-modified GCE in phosphate buffer pH 7.0: (a) Plot *I*_ox_ vs. υ for 30 µM of tartrazine (b) Plot *I*_ox_ vs. υ^½^ for 100 µM of brilliant blue FCF, Equations (S1)–(S6): Equations used for the calculation of tartrazine and brilliant blue FCF electrooxidation parameters. Figure S3: Effect of pulse parameters on the voltammetric characteristics of 10 µM mixture of tartrazine and brilliant blue FCF on the MnO_2_ nanorods-modified GCE in phosphate buffer pH 7.0: (a) the changes of peak potential separation; (b) changes in the oxidation currents of tartrazine; (c) changes in the oxidation currents of brilliant blue FCF, Figure S4: Calibration plots of dyes on the MnO_2_ nanorods-modified GCE in phosphate buffer pH 7.0: (a) Tartrazine; (b) brilliant blue FCF, Figure S5: Baseline-corrected differential pulse voltammograms for the non-equimolar mixtures of tartrazine and brilliant blue FCF on the MnO_2_ nanorods-based sensor in phosphate buffer pH 7.0: (a) various concentrations of brilliant blue FCF at the fixed 2.5 µM concentration of tartrazine; (b) various concentrations of tartrazine at the fixed 2.5 µM concentration of brilliant blue FCF. Pulse amplitude = 75 mV, pulse time = 25 ms, υ = 20 mV s^−1^, Figure S6: Baseline-corrected differential pulse voltammograms of 300 µL of real samples on the MnO_2_ nanorods-based sensor in phosphate buffer pH 7.0: (a) sample 1 with the standard additions of brilliant blue FCF; (b) sample 2 with the standard additions of tartrazine. Pulse amplitude = 75 mV, pulse time = 25 ms, υ = 20 mV s^−1^, Table S1: Recovery of tartrazine and brilliant blue FCF in real samples using MnO_2_ nanorods-based sensor in phosphate buffer pH 7.0 (*n* = 5; *p* = 0.95).
