# Supplementary material for: Selective Voltammetric Sensor for the Simultaneous Quantification of Tartrazine and Brilliant Blue FCF
Source: Sensors (Basel). 2023 Jan 17;23(3):1094. doi: 10.3390/s23031094 (PMC9920251; doi:10.3390/s23031094)
Supplement: Supplementary file 1 [file sensors-23-01094-s001.zip › sensors-2130226-supplementary.pdf]

Supplementary Materials

Selective Voltammetric Sensor for the Simultaneous Quantification of Tartrazine and Brilliant Blue FCF

Liliya Gimadutdinova, Guzel Ziyatdinova,* and Rustam Davletshin

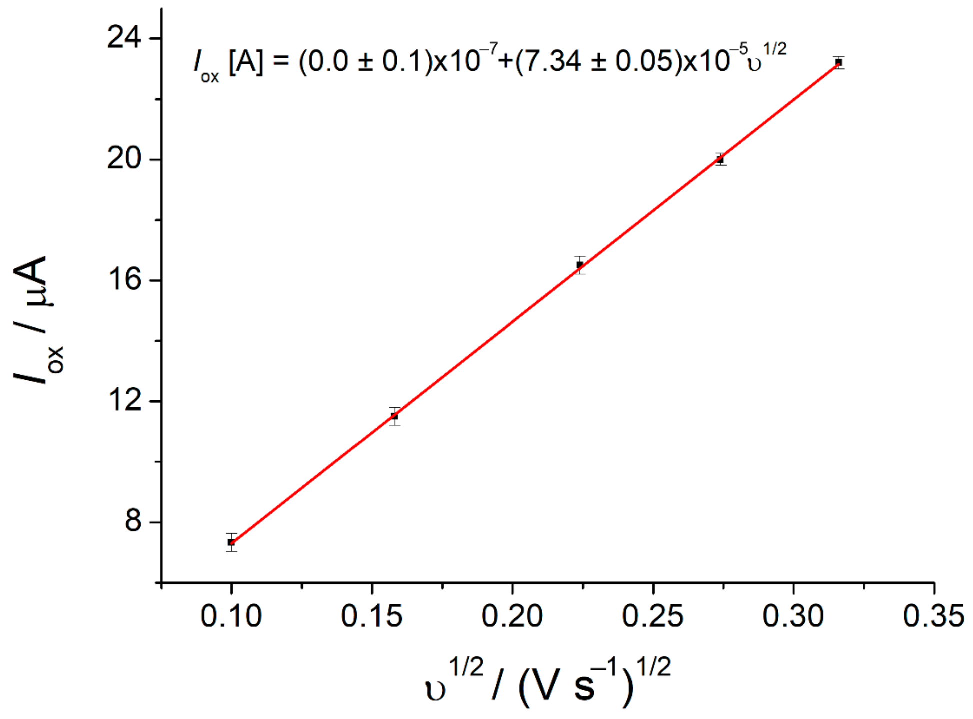

Figure S1. Plot I_{ox} vs. $υ^{½}$ for the electrooxidation of hexacyanoferrate(II) ions on the MnO_2 nanorods-modified GCE in 0.1 M KCl.

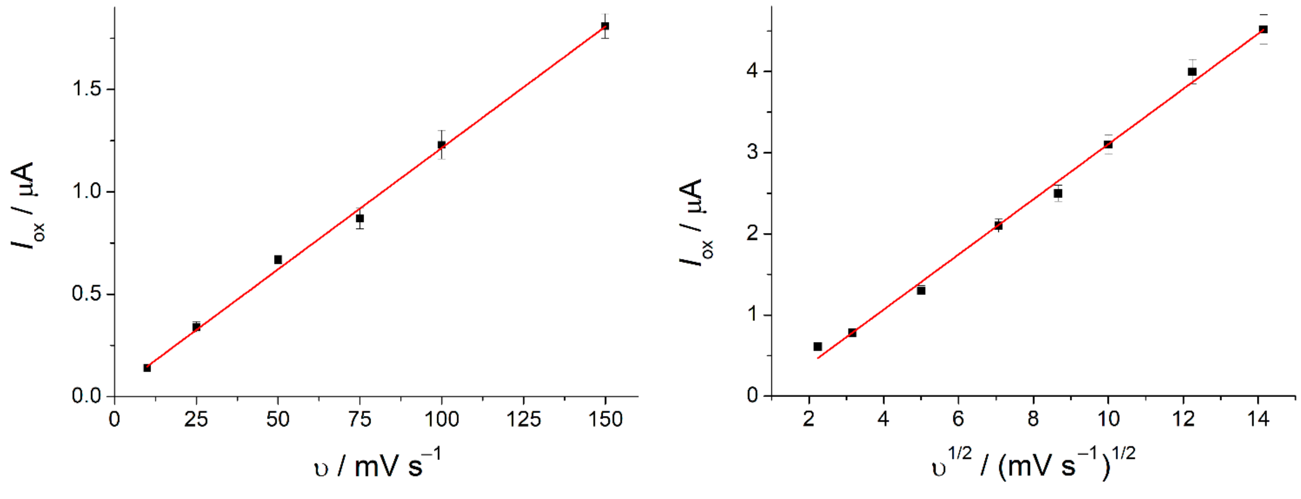

(**a**) (**b**)

Figure S2. Effect of potential scan rate on the oxidation currents of dyes on the MnO_2 nanorods-modified GCE in phosphate buffer pH 7.0: (**a**) Plot I_{ox} vs. υ for 30 μM of tartrazine (**b**) Plot I_{ox} vs. $υ^{½}$ for 100 μM of brilliant blue FCF.

Equations used for the calculation of tartrazine and brilliant blue FCF electrooxidation parameters

Laviron equation (Equation S1):

$$E_{\mathrm{ox}} = E^{0'} + \frac{RT}{\alpha_{\mathrm{a}} nF} \ln \frac{RTk_{\mathrm{s}}}{\alpha_{\mathrm{a}} nF} + \frac{RT}{\alpha_{\mathrm{a}} nF} \ln \upsilon \qquad \text{(S1)}$$

where $E^{0'}$ is the formal standard potential (V), R – universal gas constant (J mol^{-1} K^{-1}), T – temperature (K), α_a – the anodic transfer coefficient, n – the number of electrons participating in oxidation, F – the Faraday constant (C mol^{-1}), k_s – the heterogenous electron transfer rate constant (s^{-1}), υ – potential scan rate (V s^{-1}).

Surface coverage has been calculated from Equation S2

$$I = \frac{n\alpha_{\mathrm{a}} n_{\alpha} F^2 A \upsilon \Gamma}{2.718RT} \qquad \text{(S2)}$$

where n – the number of electrons participating in the oxidation, α_a – the anodic transfer coefficient, n_α – the number of electrons involved in the rate-determining step, F – the Faraday constant (C mol^{-1}), A – the electrode surface area (cm^2), υ – potential scan rate (V s^{-1}), Γ – the surface coverage (mol cm^{-2}), R – universal gas constant (J mol^{-1} K^{-1}), T – temperature (K).

Tafel plot equation for the calculation of α_a for a diffusion-controlled process:

$$slope = \frac{\alpha_{\mathrm{a}} n_{\alpha} F}{2.303RT} \qquad \text{(S3)}$$

where slope is the slope of Tafel plot log$I$ vs. E, other symbols have the same meaning as in Equation S2.

The number of electrons for the irreversible diffusion-controlled process has been calculated form Equation S4

$$\Delta E_{1/2} = 47.7/\alpha_a n \qquad \text{(S4)}$$

where $\Delta E_{1/2}$ is the difference between the oxidation potential (E) and half-wave potential ($E_{1/2}$) of brilliant blue FCF, α_a – the anodic transfer coefficient, n – the number of electrons participating in the oxidation.

Diffusion coefficien has been calculated from the Equiation S5

$$I_{ox} = \pi^{1/2} \chi(bt) nFAcD^{1/2} \left(\frac{\alpha_{\mathrm{a}} n_{\alpha} F}{RT}\right)^{1/2} \upsilon^{1/2} \qquad \text{(S5)}$$

where I_{ox} – oxidation peak current (A), $\chi(bt)$ – normalized current for sweep experiments with an irreversible system, n – the number of electrons participating in oxidation, F – the Faraday constant (C mol^{-1}), A – the electrode surface area (cm^2), c – concentration (mol cm^{-3}), D – diffusion coefficient (cm^2 s^{-1}), α_a – anodic transfer coefficient, n_α – the number of electrons involved in the rate-determining step, R – the gas constant (J mol^{-1} K^{-1}), T – temperature (K) and υ – potential scan rate (V s^{-1}).

The standard heterogeneous electron transfer rate constant k^0 has been calculated using Equation S6

$$k^0 = 2.415 e^{-\frac{0.02F}{RT}} D^{1/2} (E - E_{1/2})^{-1/2} \upsilon^{1/2} \qquad \text{(S6)}$$

where k^0 is the standard heterogeneous electron transfer rate constant (cm s^{-1}), D – diffusion coefficient (cm^2 s^{-1}), E – the oxidation potential (V), $E_{1/2}$ – the oxidation half-wave potential (V), other symbols have the same meaning as in Equation S2.

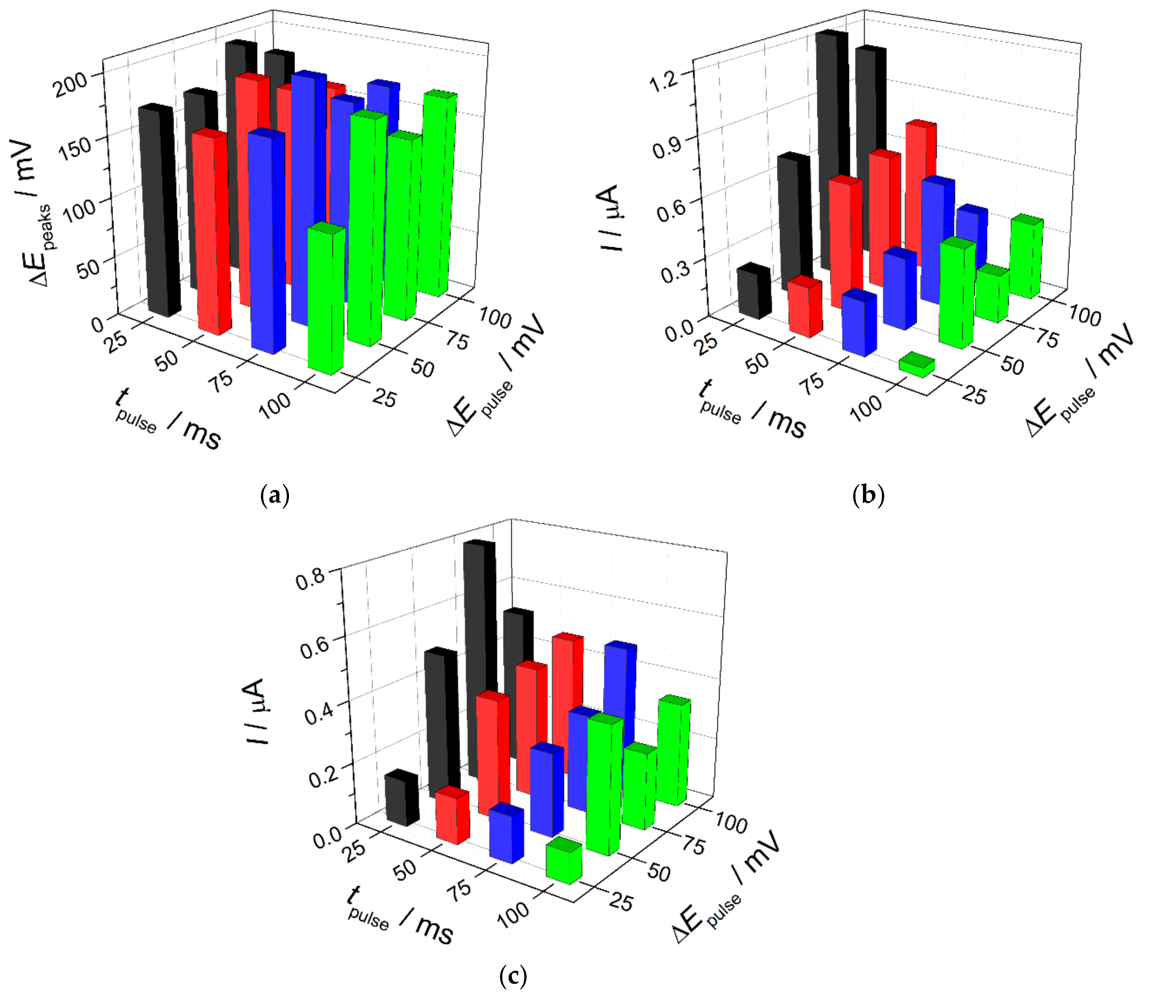

Figure S3. Effect of pulse parameters on the voltammetric characteristics of 10 µM mixture of tartrazine and brilliant blue FCF on the MnO_2 nanorods-modified GCE in phosphate buffer pH 7.0: (**a**) the changes of peak potential separation; (**b**) changes in the oxidation currents of tartrazine; (**c**) changes in the oxidation currents of brilliant blue FCF.

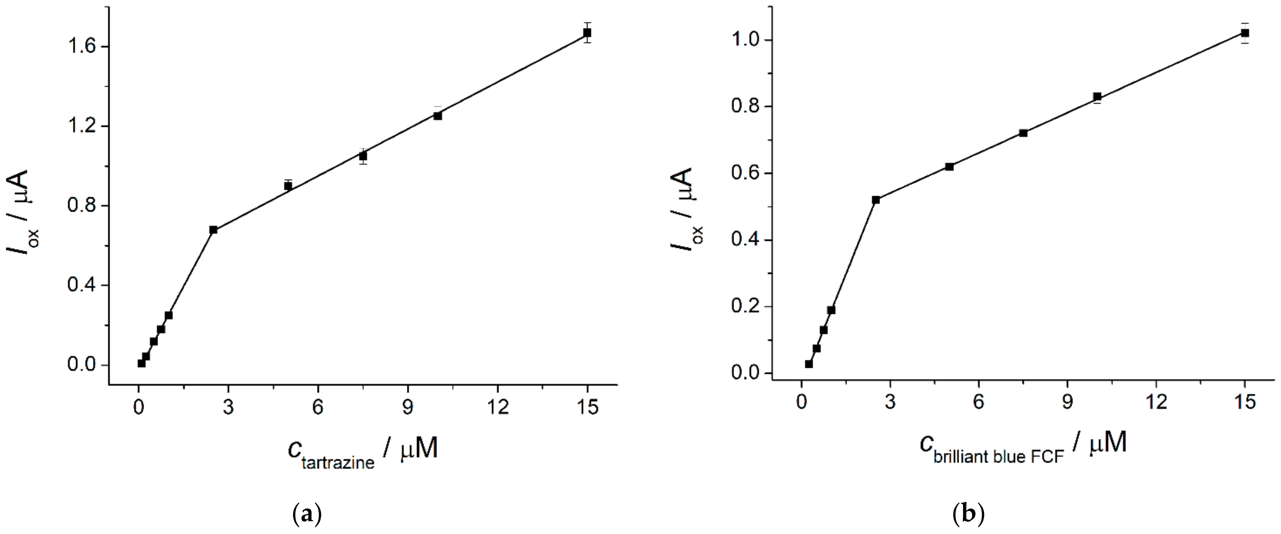

Figure S4. Calibration plots of dyes on the MnO_2 nanorods-modified GCE in phosphate buffer pH 7.0: (**a**) Tartrazine; (**b**) brilliant blue FCF.

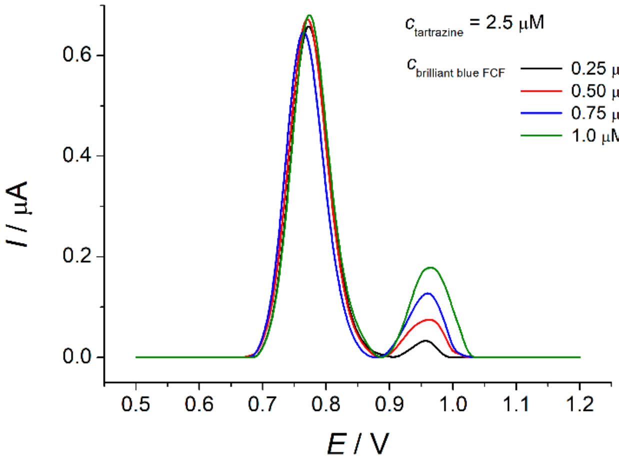

(**a**)

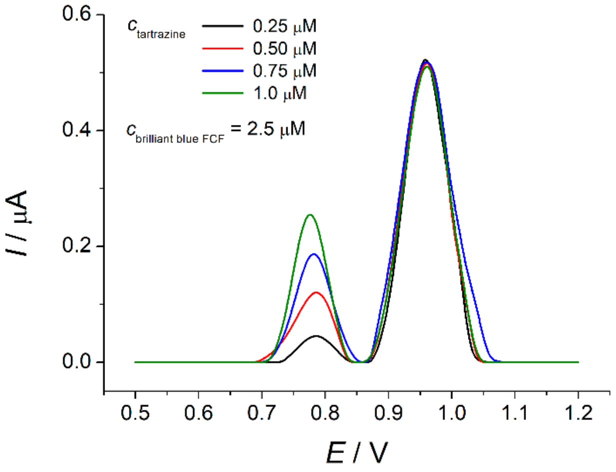

(**b**)

Figure S5. Baseline-corrected differential pulse voltammograms for the non-equimolar mixtures of tartrazine and brilliant blue FCF on the MnO_2 nanorods-based sensor in phosphate buffer pH 7.0: (**a**) various concentrations of brilliant blue FCF at the fixed 2.5 μM concentration of tartrazine; (**b**) various concentrations of tartrazine at the fixed 2.5 μM concentration of brilliant blue FCF. Pulse amplitude = 75 mV, pulse time = 25 ms, υ = 20 mV s^{-1}.

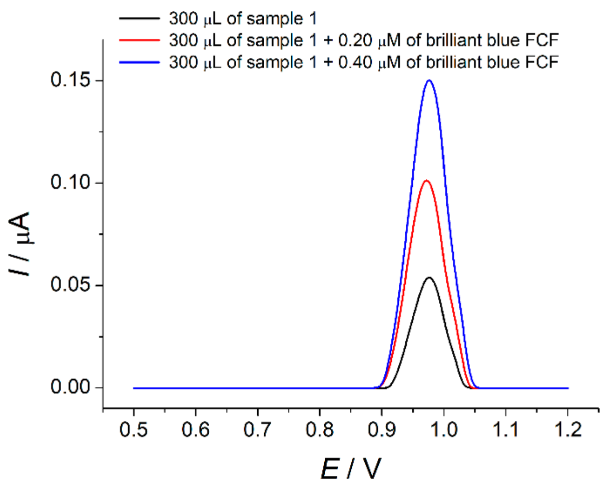

(**a**)

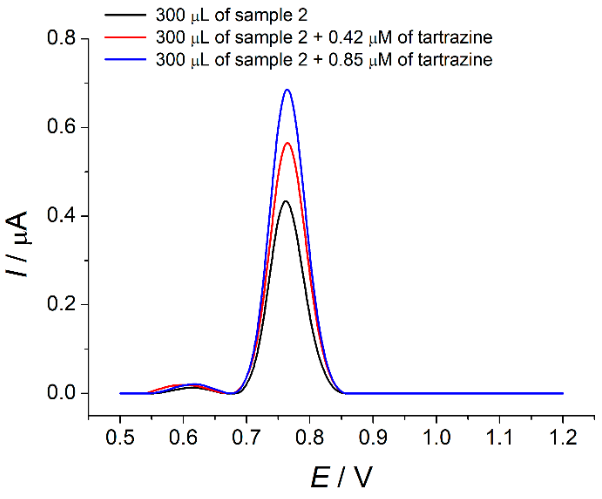

(**b**)

Figure S6. Baseline-corrected differential pulse voltammograms of 300 μL of real samples on the MnO_2 nanorods-based sensor in phosphate buffer pH 7.0: (**a**) sample 1 with the standard additions of brilliant blue FCF; (**b**) sample 2 with the standard additions of tartrazine. Pulse amplitude = 75 mV, pulse time = 25 ms, υ = 20 mV s^{-1}.

Table S1. Recovery of tartrazine and brilliant blue FCF in real samples using MnO_2 nanorods-based sensor in phosphate buffer pH 7.0 (n = 5; P = 0.95).

Sample	Dye	Spiked (μM)	Found (μM)	RSD (%)	*R* (%)
Sample 1	Brilliant blue FCF	0	0.40 ± 0.01	2.5	
		0.20	0.60 ± 0.02	2.4	100 ± 3
		0.40	0.80 ± 0.02	2.3	100 ± 2
Sample 2	Tartrazine	0	1.68 ± 0.07	3.3	
		0.42	2.09 ± 0.07	2.8	99 ± 3
		0.85	2.55 ± 0.09	2.2	100 ± 2
